# Utility of time-lapse photography in studies of seabird ecology

**DOI:** 10.1371/journal.pone.0208995

**Published:** 2018-12-12

**Authors:** Federico De Pascalis, Philip M. Collins, Jonathan A. Green

**Affiliations:** 1 Department of Life Sciences, University of Trieste, Trieste, Italy; 2 School of Environmental Sciences, University of Liverpool, Liverpool, United Kingdom; 3 School of Life Sciences, University of Roehampton, London, United Kingdom; MARE – Marine and Environmental Sciences Centre, PORTUGAL

## Abstract

Marine ecosystems are heavily influenced by a wide range of human-related impacts, and thus monitoring is essential to preserve and manage these sensitive habitats. Seabirds are considered important bioindicators of the oceans, but accessing breeding populations can be difficult, expensive and time consuming. New technologies have been employed to facilitate data collection on seabirds that can reduce costs and minimize disturbance. Among these, the use of time-lapse photography is a potentially effective way to reduce researcher effort, while collecting valuable information on key ecological parameters. However, the feasibility of this approach remains uncertain. Here, we assessed the use of time-lapse photography as a tool for estimating foraging behaviour from breeding seabirds, and evaluate ways forward for this method. We deployed cameras in front of active nests at a colony of black-legged kittiwakes (*Rissa tridactyla*) during two breeding seasons, 5 nests in 2013 and 5 in 2014, taking pictures every 4 minutes. A subsample of monitored individuals were also equipped with accelerometers. Approximately 100,000 frames, covering incubation and chick-rearing periods, were analysed. Estimates of foraging trip duration from images were positively correlated with accelerometry estimates (R^2^ = 0.967). Equal partitioning of effort between pairs, predation events, nest attendance patterns and variation in trip metrics with breeding stage were also identified. Our results suggest that time-lapse photography is potentially a useful tool for assessing foraging trip duration and other fine-scale nesting ecology parameters as well as for assessing the effect of bio-logging devices on seabird foraging behaviour. Nevertheless, the time investment to manually extract data from images was high, and the process to set up cameras was not straightforward. To encourage wide use of time-lapse photography in seabird ecology, we thus provide guidelines for camera deployment and we suggest a need for further development of automated approaches to allow data extraction.

## Introduction

The world’s marine ecosystems are experiencing biological change at an alarming rate, owing largely to human driven pressures [1–4]. Consequently, there is an urgent need to identify and utilize tools, such as bioindicators, that can help to assess ecosystem health and meet conservation objectives [5]. Seabirds have been proposed, and indeed used, as suitable bioindicators in the marine environment, due to their position as top consumers within marine foodwebs [6], their sensitivity to variations in food supply [6–8], and their behaviour as central-place foragers during the breeding season [9].It has long been proposed that immediate changes in forage fish distribution and abundance can be detected in foraging effort of breeding adult seabirds [7], which has often been measured by length of time an adult is absent from the nest [10]. Time spent foraging is predicted to increase with poor food supplies [7], and hence the relationship between measurements of foraging activity and food availability is a potentially informative metric when using seabirds as bioindicators. [7]. Foraging trip duration and levels of nest attendance have been strongly linked to prey availability in species that demonstrate a wide range of feeding techniques [8, 10, 11–14]. For surface feeding species such as black-legged kittiwakes (*Rissa tridactyla*), which use only the top few meters of the water column, reduced prey availability can only be compensated for by intensifying search effort and/or increasing foraging range and trip duration [10]. Therefore for these species, foraging trip duration is potentially a particularly sensitive and powerful parameter for assessing changes in prey availability, which may in turn be indicative of wider change in ecosystems. However, as seabirds are highly mobile animals and often breed in isolated and remote populations which are difficult and expensive to reach, trip duration is often difficult to measure [15–17].

The current most widespread technique for studying seabird foraging behaviour is biologging [18] but there are certain limitations to this approach. Notably, devices are likely to have a negative impact on the instrumented animal, which may in turn affect the parameters being measured [19]. For example tagging breeding birds can result in a lower provisioning rate or may induce a partner to take on a greater burden of chick-provisioning effort [20–22]. In recognition of the potential impact of tags, deployment duration tends to be short, limiting how much of an entire breeding season can be studied. As a result, this limits the number of foraging trips that can be recorded. In order to have enough trips to draw reliable conclusions, then the number of devices deployed has to be increased, which in turn increases the financial expenditure and effort in the field, while still limiting the investigation to a short temporal frame.

A potential approach to mitigate these problems is the use of time-lapse photography to record foraging trip durations and behaviours at the nest. There is a growing interest in the use of cameras in ornithology [23], and advances in digital technology have led to increased storage capacity, faster and easier review of data, less frequent maintenance and reduced power consumption [24]. The use of remote time-lapse photography has been recognised as a valuable way to record baseline ecological data such as breeding productivity, while reducing researcher effort [17]. However, this approach also has the potential to simultaneously assess multiple important aspects of seabird behaviour and ecology at a finer scale, such as foraging trip duration, frequency of foraging trips, nest attendance, timing of changeovers, division of labour between parents and predation events. This may allow us to gather important information about health status of the colony (and therefore about the environment) and about behavioural adaptations to variable extrinsic conditions over an entire breeding season, but the utility of this approach has yet to be tested extensively [25].

The aim of our work was (i) to test whether time-lapse photography is a feasible method for obtaining reliable information on foraging trip duration and (ii) to test the utility of time-lapse photography to simultaneously assess different parameters of seabird breeding ecology, including predation, attendance patterns and effect of disturbance. In light of our findings and experience we also provide recommendations on the steps needed to further develop this approach.

## Materials and methods

### Ethic statement

All work was conducted under Licence by the Countryside Commission for Wales (2013: 44043:OTH:SB:2013) and Natural Resources Wales (2014: 53628:OTH:SB:2014) following the provisions of the Wildlife and Countryside Act 1981. Permission to work on our privately-owned study site was granted by Sir Richard Williams-Bulkeley and the Baron Hill Estate. All tagged birds were handled by trained individuals following guidelines developed by the British Trust for Ornithology [26].

### Study area and species

The study was conducted at a breeding colony of black-legged kittiwakes (*Rissa tridactyla*) on Puffin Island, a Special Protection Area (SPA) with an area of 0.31 Km^2^, located off North Wales, UK (Latitude 53°19΄02˝N, Longitude 04°01΄36˝W). The island hosts breeding populations of ten seabird species, including approximately 300 pairs of black-legged kittiwakes (*Rissa tridactyla*). The black-legged kittiwake (hereafter kittiwake) is a small gull widely distributed in temperate and Arctic regions in the Northern Hemisphere. During spring and summer kittiwakes breed in dense colonies on sea cliffs, and spend the remainder of the year distributed across the northern oceans [27]. Since the late 1980s, they have been the subject of a United Kingdom-wide programme to monitor annual breeding success and population size [28, 29] that has highlighted a marked population decline since the early 1990s [28], and due to their ongoing global decline, they have been recently uplisted to “Vulnerable” by the IUCN [30]. This decline is thought to be driven by low reproductive success, which has been primarily attributed to reduced food availability [30–33]. They often rely on few prey species such as sand lance (*Ammodytes* spp.), capelin (*Mallotus villosus*) and juvenile cods (Gadidae) [10, 27, 29] and have a limited capacity to switch to alternative prey due to their surface-feeding habits. Because of these factors, kittiwakes are considered a good indicator species of fluctuations in physical and trophic conditions [29, 31] in the marine environment and are often used as bioindicators [34].

### Cameras

#### Deployment

Camera-traps (Ltl-Acorn 5210MC, costing ~US$ 116 per camera) were deployed within the kittiwake colony at the beginning of the 2013 (4 cameras) and 2014 (7 cameras) incubation periods and left until the late chick rearing periods. Each camera was connected to an external lead acid battery (12V 7Ah), and batteries were changed once after 4 weeks. Cameras were set to record pictures (12-megapixels) every 4 minutes (time-lapse mode) during both day and night. This sampling rate was chosen in order to obtain a balance between good temporal resolution and a long battery life. They were deployed at a distance between 2 and 15 meters and with at least 4 nest visible in each camera’s field of view. During the night, images were recorded using the Passive Infra-Red (PIR) sensor. Our aim was to capture the majority of the incubation and entire chick-rearing period for all nests within the field of view of the cameras. However, cameras occasionally suffered from brief malfunctions or erroneous set-ups, leading to some gaps in the data. To aid identification of pair members in the camera data, 29 birds across both years of study (one per nest) were caught using a nylon noose and pole, and marked on both the head and breast with orange picric acid dye.

#### Data extraction

Approximately 100 000 frames, spanning the incubation and chick-rearing periods of 10 nests (*n*, 2013 = 5, 2014 = 5) and 20 individuals were inspected visually, covering on average 665 hours per nest. The data-extraction phase required more than 90 hours of observer work.

For each frame for each study nest, an observer recorded the number of birds and their identity (marked or unmarked), reproductive status (incubation or chick-rearing), number and age of chicks (0 = day of hatching), and presence/absence of accelerometers (see ‘validation’ below). Predation events at nests, the date and time of each departure/arrival of birds from nest sites, and the presence of data gaps due to camera malfunctions were also noted. During night-time, it was not possible to detect colour marks on birds, and therefore changes in bird identity at the nest were inferred by checking body movements and positional differences noted during daytime changes and through repeat checks of successive frames in sequence.

Secondary parameters relating to diurnal and nocturnal behaviours were inferred from these data (Table 1). These included time spent away from the nest, frequency of nest departures, nest attendance and an index for the number of trips, accounting for the multiple gaps in the series of frames due to camera malfunctions. To account for disturbance caused by potential predators, we noted whether humans or peregrine falcons (*Falco peregrinus*; hereafter referred to as peregrine) were at, or nearby, nest sites for each departure/arrival event, and the time away from the nest was calculated. Human-induced disturbance was defined as a human passing close to the nest, either on the cliff’s edge (i.e. our study team or licensed bird ringers) or from the sea (i.e. kayaks, sea-watching tours). Peregrine disturbance was defined as a kittiwake leaving the nest site with a peregrine perched on or close to the nest. All of the listed disturbances were visible in the camera frames. Based on the duration of periods away from the nest caused by disturbance (“scared trip”), a foraging trip was defined as a period away from the nest lasting more than 25 minutes. This minimum threshold helped us to discard trips that could have been caused by natural or human disturbance, but were not detected with cameras due either to the disturbance being outside of the camera’s frame of vision or occurring within the sampling interval between images being captured.

**Table 1:** Parameters (and their related estimators) inferred from camera data for quantifying kittiwake behaviours at the Puffin Island colony, North Wales.

Parameter	Estimator
**Time away from nest**	time of arrival—time of previous departure from nest
**Nest departure frequency**	$\frac{n{}^{\circ}\;of\;departures\;at\;time\;t}{n{}^{\circ}\;of\;total\;departures}$
**N° of trip index**	$\frac{total\;n{}^{\circ}\;of\;trips}{period\;recorded\;(minutes)}\times 10^{4}$
**Nest attendance**	$\frac{n{}^{\circ}\;of\;frames\;with\;0,1,2\;birds}{total\;number\;of\;frames}\%$

#### Validation

To test if camera-derived time spent away from nest was a good measure of foraging trip duration, a subset of the data was used which incorporated instances during the chick-rearing period when some of the birds (2 from 2013 and 3 from 2014) were equipped for 2–3 days with a tri-axial accelerometer (X8 m-3 Gulf Coast Data Concepts, LLC; frequency of recordings: 50 Hz, recording range: 8 g, resolution: 0.001 g, weight: 14 g). See [35, 36] for more details of deployment procedures, processing of accelerometer data and extraction of behaviours. For these five birds, foraging trip durations were recorded using both the accelerometers and camera images.

All subsequent analyses were conducted in R (version 3.2.4) [37]. The mean difference in trip duration between the two recording methods was tested with a linear mixed effects model (LMM) using the ‘nlme’ package [38]. Recording method (‘camera’ or ‘accelerometer’) was fitted as a fixed effect, and ‘bird identity’ was modelled as a random intercept. A post-hoc Tukey test was performed on least-squares means (accounting for unbalanced sampling design). To test if the parameter “time away from nest” recorded with cameras was a good proxy of the foraging trip duration (recorded with accelerometers), a Spearman correlation test was performed at the individual foraging trip level between trips measured both with cameras and accelerometers.

### Camera data analysis

To investigate the influence of breeding stage (fixed effect, ‘incubation’ or ‘chick rearing’) and to analyse changes over time (fixed effect: chick age, a discrete variable where 0 is the day of hatching, positive values are the number of days after hatching and negative values the number of days before hatching) on foraging trip duration, generalized linear mixed effects models (GLMMs) were applied to the full camera dataset. GLMMs were also run on the subset of images with matching bio-logging data to analyse the effect of accelerometer presence/absence (fixed effect) on trip duration. To account for repeated measures from individual birds and nests, ‘bird identity’ was included as a random intercept, nested in a second random effect for ‘nest identity’. Models were constructed using a gamma error family distribution with a logarithmic link. In some cases, a post-hoc Tukey test was performed on least-squares means. All models were constructed using the ‘lme4’package [39], and when *P*-values were close to 0.05 the most parsimonious model was also fitted using the glmmPQL function from the ‘MASS’ package [40]. To investigate differences in foraging between members of a pair, the number of trips per animal and foraging trip duration were compared using a non-parametric Wilcoxon rank sum test.

## Results

### Camera failures

A large number of failures happened, leading to the study incorporating fewer nests than expected. The high failure rate was both due to natural factors (i.e. 8 nests failed for natural reasons during incubation just after the start of the monitoring in what was a poor breeding season) and to camera failures, such as errors in the camera set-ups (Table 2). Monitoring foraging trip duration with cameras requires high-resolution data where it is always possible to check the identity of the bird present at nest. Data where those high-quality standards were not met were discarded, in order not to bias the results with inaccurate foraging trip durations.

**Table 2:** Camera deployment details for 2013 and 2014 breeding seasons from Puffin Island, North Wales. The table shows the number of visible nests, the number of nests with marked birds in the sample and the effective number of birds used for analysis. Failures and reasons for exclusion from the final data set are also displayed.

	Camera n°	Nests visible (n°)	Nests with marked birds (n°)	Nests with used birds (n°)	Reason of failure
**2013**	1	6	2	0	Camera too far
	2	9	5	1	Nests failed after a few days
	3	4	0	0	
	4	5	4	4	
**2014**	1	9	1	0	Camera too far
	2	7	3	2	Wrong angle for 3^rd^ nest
	3	4	2	0	Wrong angle
	4	5	4	0	Wrong angle + nest failure
	5	4	2	0	Wrong angle + camera too far
	6	9	3	0	Wrong angle + camera too far + backlight
	7	8	3	3	

### Camera data validation

**Fig 1:**
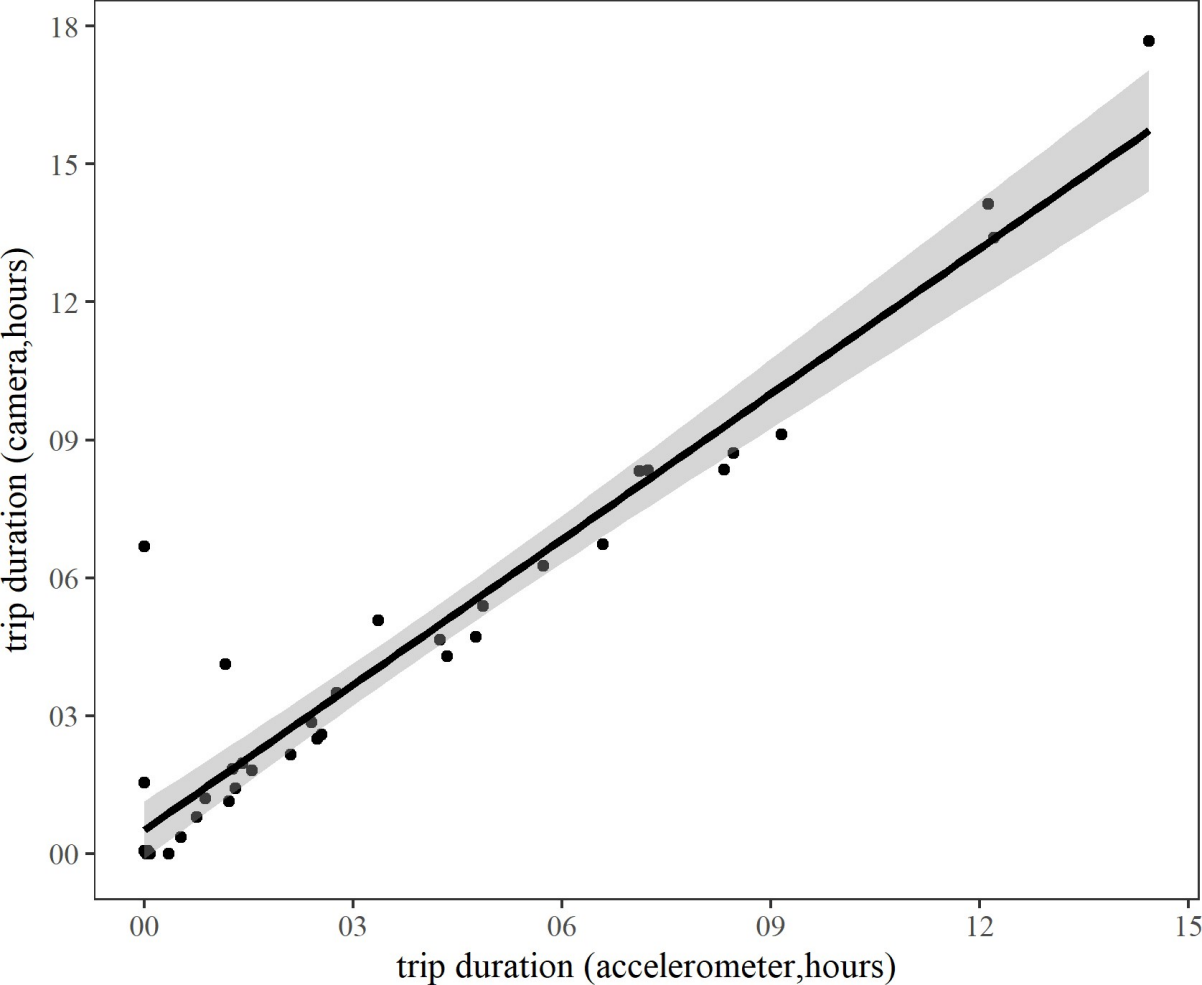
Time away from nest as a proxy of foraging trip duration. Correlation between time away from nest (recorded with cameras) and foraging trip duration (recorded with accelerometers) of kittiwakes from Puffin Island, North Wales. 95% confidence interval is shown in grey.

There was no significant difference in least square means between trip duration recorded using accelerometers and time away from the nest recorded using cameras (LMM, LS mean ± SE: Accelerometer = 328 ± 93 mins, Cameras = 376 ± 93 mins, t = -1.04, *P =* 0.30). The two measures were highly correlated, and the time away from nest recorded using cameras is a good proxy for foraging trip duration (Fig 1, Spearman correlation coefficient = 0.967, *P<0*.*001*) and therefore we used this measure in subsequent analyses.

### Behaviour at nest

#### Nest attendance

**Fig 2:**
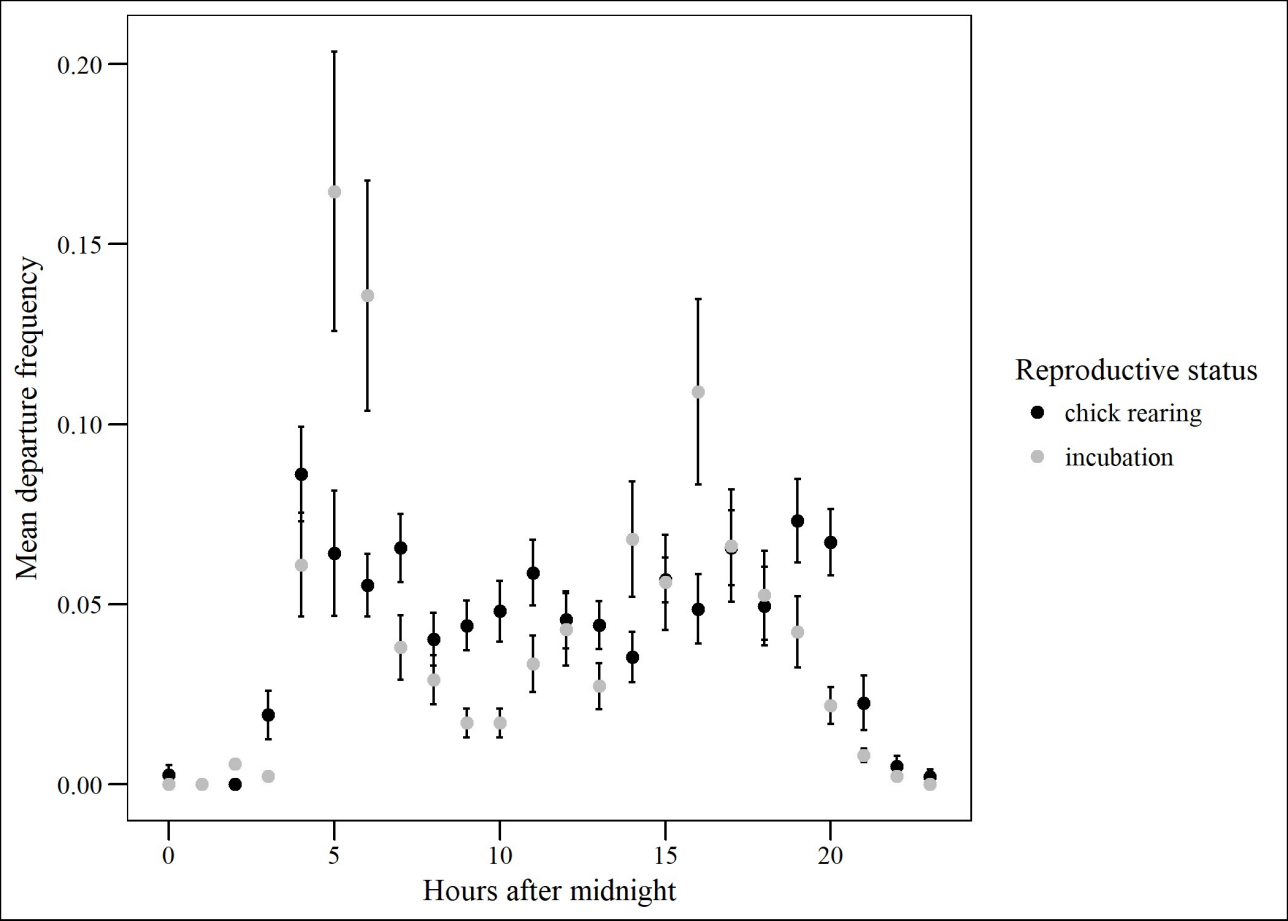
Nest departure frequency of kittiwakes from Puffin Island, North Wales. Mean departure frequency **(**± SE**)** per hour of the day during incubation and chick-rearing periods.

During incubation, there was a peak of nest departures in early morning (04.00–06.00) and late afternoon (15.00–17.00), while during chick-rearing, peaks in departure times were less pronounced (Fig 2). This pattern was consistent, with little variability between individuals (S1 Fig). A similar pattern was detected in arrival times, as expected due to patterns of nest attendance and pair changeovers. In almost all cases, one pair member was in nest attendance at any given time. During chick rearing, one pair member was present for 97.5% of the time, two were present for 0.8% of the time and nests were unattended for 1.7% of the time. During incubation, nests were left unattended slightly less frequently, however the proportion of time with both adults recorded on the nest was slightly higher: one adult was present for 99.5% of the time, two present for 0.1% of the time and no adults present for 0.4% of the time.

#### Predation

**Fig 3:**
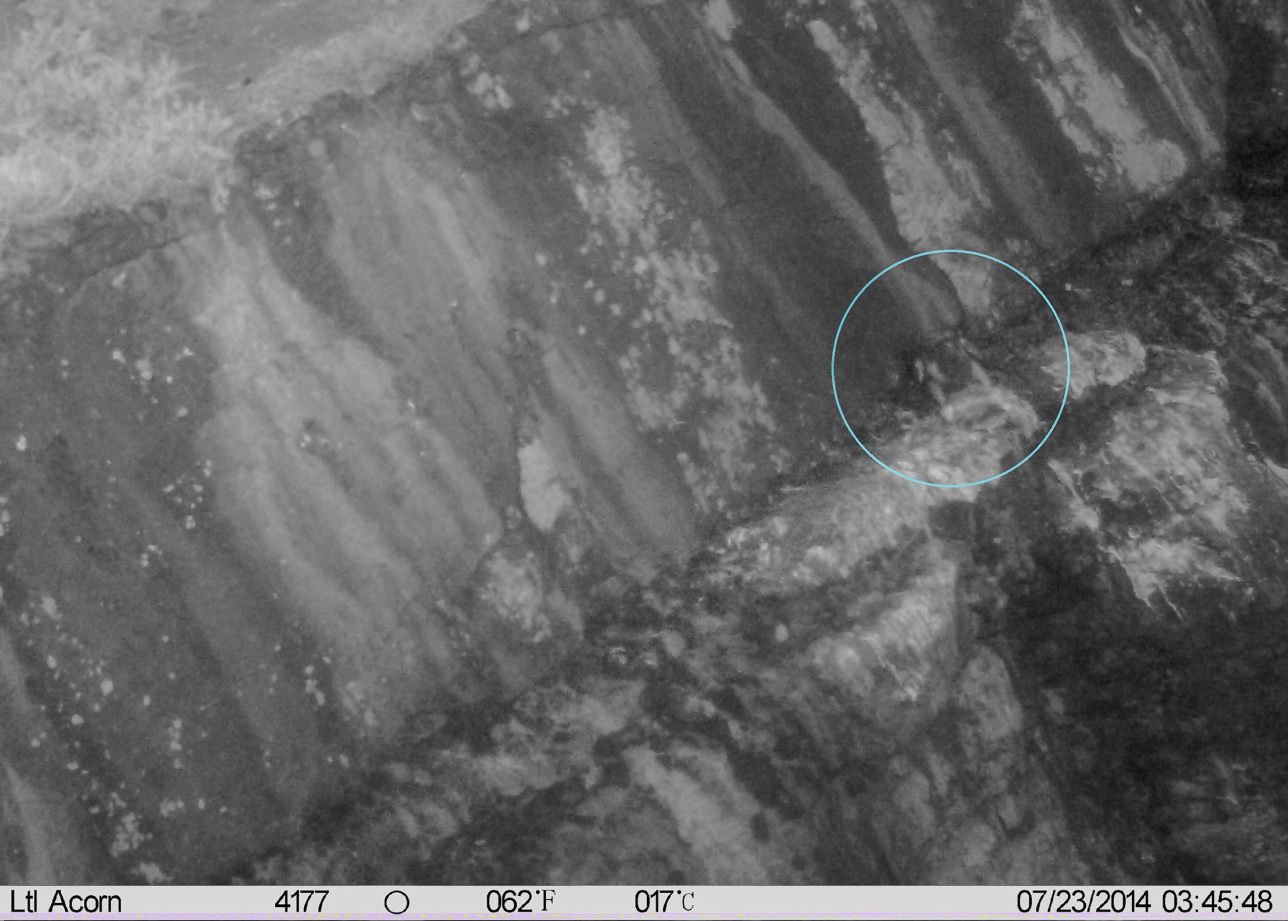
Example camera image showing night predation at kittiwake nest at the Puffin Island colony, North Wales. Peregrine falcon predation event on a chick at night, during the 2014 breeding season is shown. Time and date are displayed, and peregrine is indicated with a blue circle.

During the chick-rearing phase, four of the ten study nests were positively identified as predated by peregrines during night-time (Fig 3), and a further four nests were likely predated by the same species. In these cases, the predation event was not directly recorded, however, the sudden failure of the nest during night, an absence of the parents and the proximity of the nests to those that had been recently predated were also suggestive of peregrine predation as the cause of nest failure. One nest was predated by a herring gull (*Larus argentatus*).

**Fig 4:**
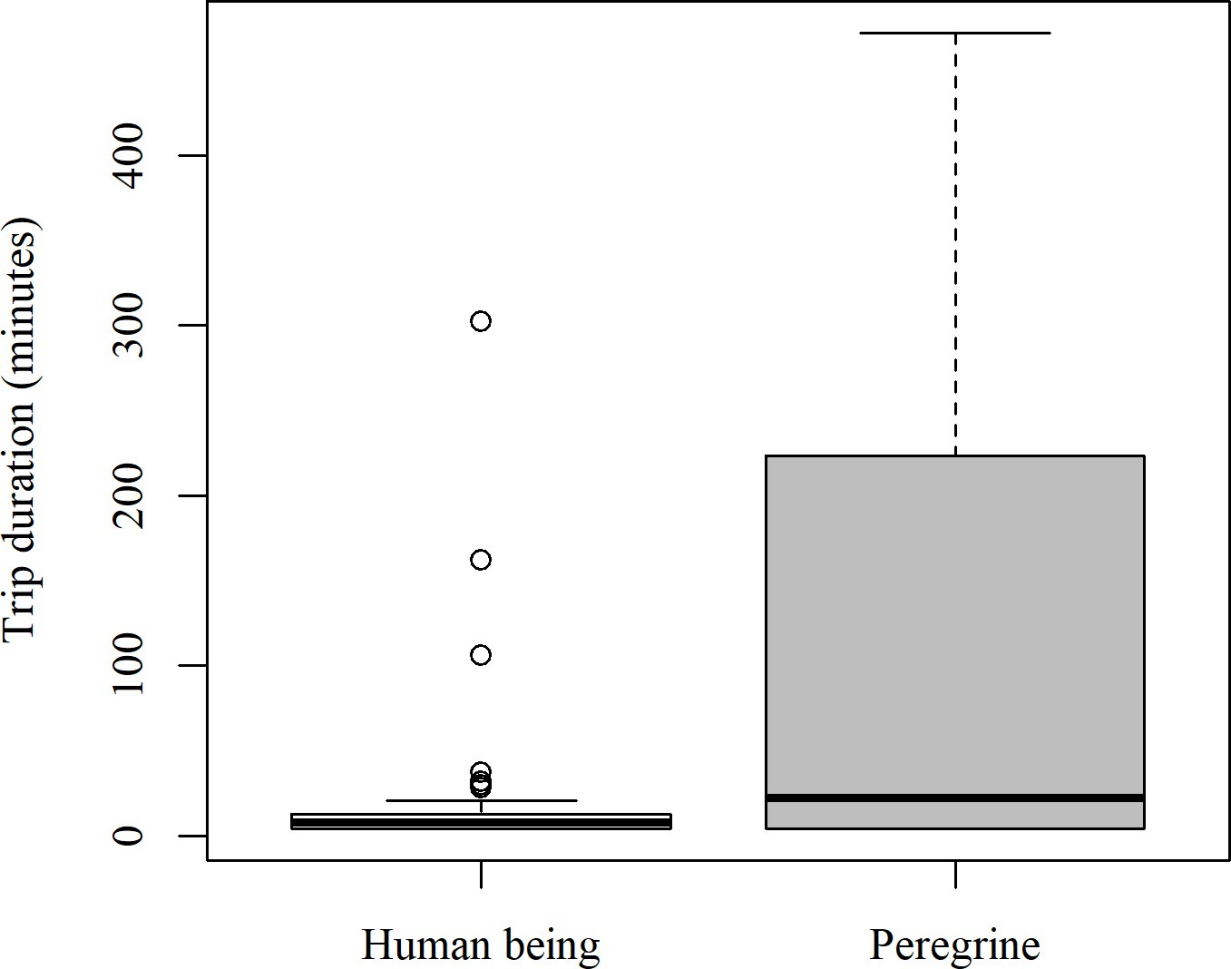
Length of “scared trips” in kittiwakes from Puffin Island, North Wales. Differences in trip duration of kittiwakes when scared by humans and peregrines.

There was no significant difference in mean trip duration for “scared trips” resulting from peregrine presence (mean ± SE:119 ± 52 mins) or human disturbance (20 ± 6 mins) (Wilcoxon rank sum test, *W* = 240.5, *P* = 0.124; Fig 4). However, it should be noted that sample size for this analysis is small (*n* = 12 and 59 trips, respectively) and hence the power of the test is low.

### Behaviour away from nest

**Fig 5:**
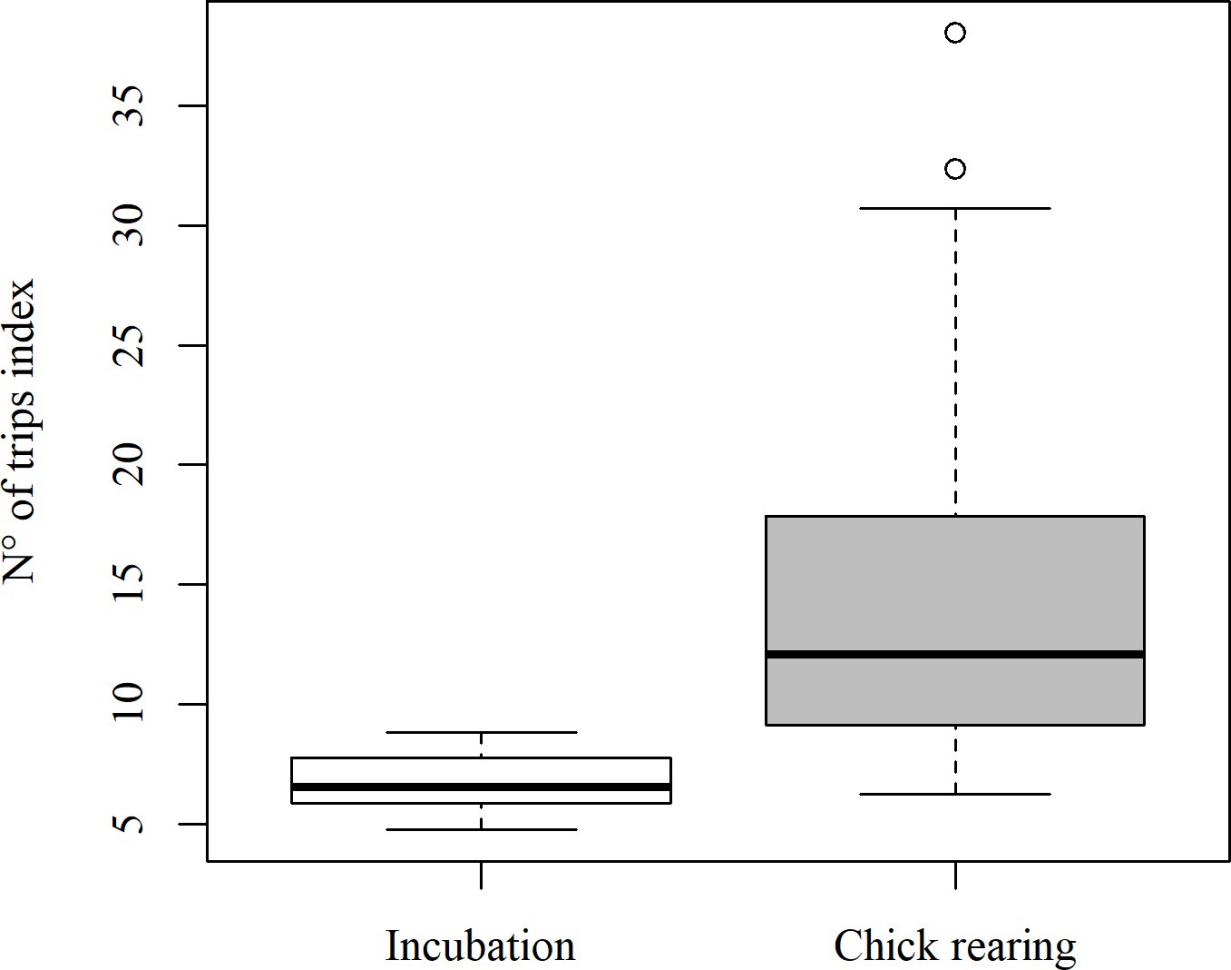
Number of foraging trips of kittiwakes from Puffin Island, North Wales. The index, accounting for gaps in camera data, shows differences in number of foraging trips between incubation and chick-rearing periods.

**Fig 6:**
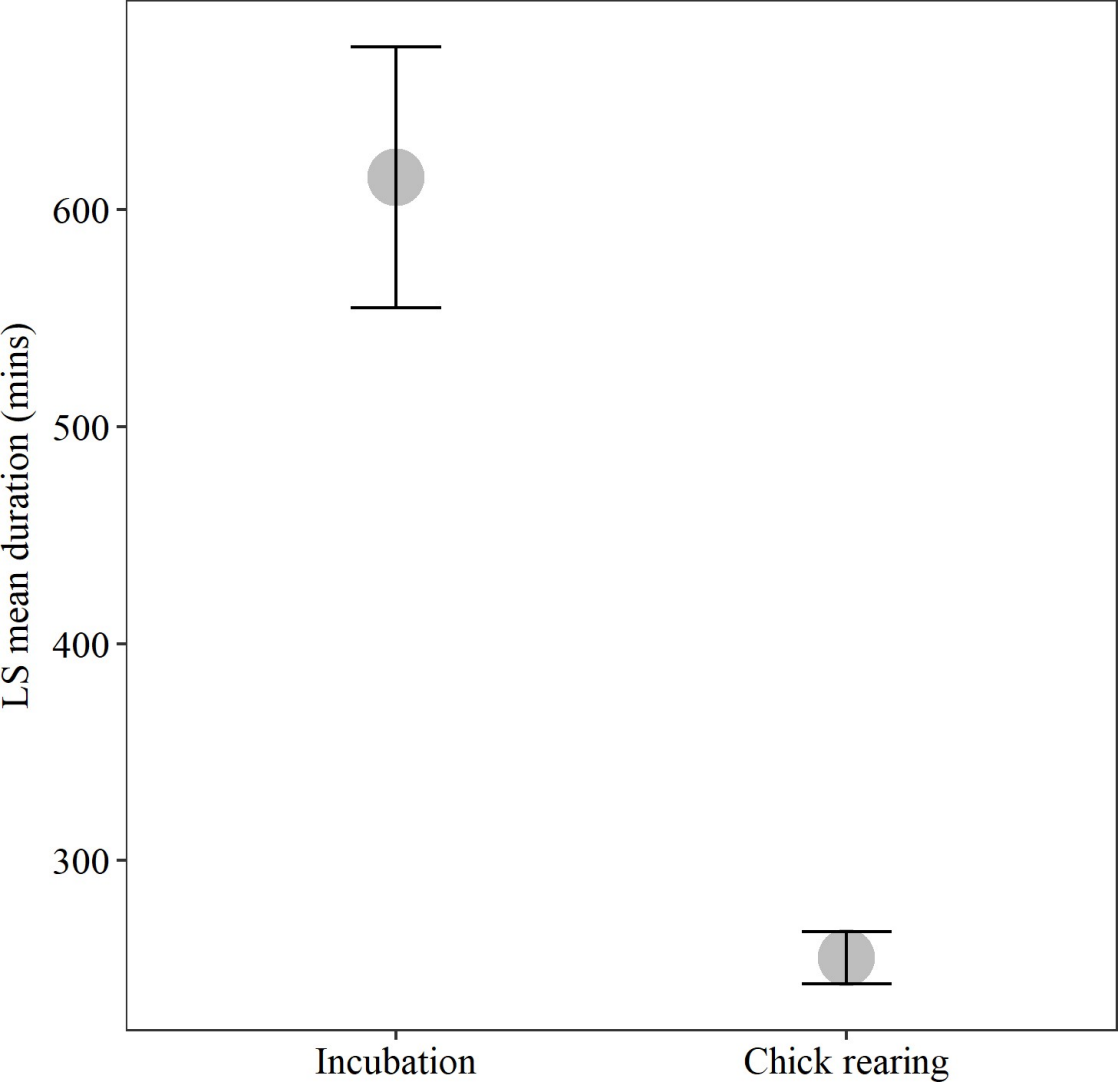
Foraging trip duration during different breeding stages for kittiwakes from Puffin Island, North Wales. Least squares means (± SE) of foraging trip durations from the fitted GLMM model are shown during incubation and chick rearing.

A total of 850 foraging trips (289, 561 during incubation and chick rearing respectively) were recorded from camera data, and there were significantly fewer trips during incubation than during chick rearing (Wilcoxon rank sum test, *W* = 292, *P* < 0.001; Fig 5). Foraging trip duration was significantly shorter during the chick-rearing period (GLMM, LS mean ± SE: Incubation = 614 ± 60 mins, Chick rearing = 255 ± 12 mins, t = -17.02, *P*<0.001; Fig 6). There was no strong evidence for a systematic change in foraging trip duration within these two periods for either model when fitted with both penalized quasilikelihood and Laplace approximations (Table 3). There was no evidence for an effect of accelerometer attachment on foraging trip duration (GLMM, LS mean ± SE: Off = 244 ± 23 mins, On = 261 ± 35 mins, t = 0.65, *P =* 0.52). There was no evidence for a difference in the number of foraging trips (Table 4) between pair members. There was no evidence for a systematic difference in foraging trip duration (Table 5) between pair members (only 3 pairs out of 8 showed some differences in foraging trip duration).

**Table 3:** Model parameters from fitted GLMMs models used to test the influence of reproductive status and chick age on kittiwake foraging trip durations at the colony on Puffin Island, North Wales.

Reproductive status	GLMM Model parameters	Estimated coefficient	Significance level	SE	Parameter estimation method
**Incubation**	**Intercept**	6.42	<<0.05	0.10	Laplace approximation
**Incubation**	**Chick age**	-0.01	0.38	0.01	Laplace approximation
**Chick rearing**	**Intercept**	5.43	<<0.05	0.09	Laplace approximation
**Chick rearing**	**Chick age**	0.01	0.07	0.01	Laplace approximation
**Incubation**	**Intercept**	6.42	0.00	0.09	PQL
**Incubation**	**Chick age**	-0.01	0.31	0.00	PQL
**Chick rearing**	**Intercept**	5.43	0.00	0.09	PQL
**Chick rearing**	**Chick age**	0.01	0.07	0.01	PQL

**Table 4:** Summary of the number of foraging trips recorded by the cameras of individual kittiwakes from Puffin Island, North Wales, during incubation and chick-rearing periods in 2013 and 2014.

Nest id	Individual	N° of trips (incubation)	N° of trips (Chick rearing)
1 1	A1	20	26
	B1	19	21
**2**	A2	NA^a^	27
**2**	B2	NA^a^	30
**3**	A3	10	37
**3**	B3	8	33
**4**	A4	13	35
**4**	B4	14	36
**5**	A5	26	26
**5**	B5	25	28
**6**	A6	4	NA^b^
**6**	B6	7	NA^b^
**7**	A7	14	49
**7**	B7	12	58
**8**	A8	16	18
**8**	B8	14	17
**9**	A9	20	24
**9**	B9	19	24
**0**	A10	25	35
**0**	B10	23	33

^a^ The camera was deployed at the beginning of the chick rearing period.

^b^The chick was predated the day after hatching

**Table 5:** Differences in foraging trip duration during incubation and chick-rearing periods from kittiwakes on Puffin Island, North Wales.

Nest id^a^	W	P value	Status
**1** **1**	222	0.38	Incubation
	212	0.20	Chick rearing
**3**	23	0.14	Incubation
**3**	486.5	0.15	Chick rearing
**4**	73	0.39	Incubation
**4**	505	0.15	Chick rearing
**5**	291	0.53	Incubation
**5**	427.5	0.27	Chick rearing
**7**	65	0.35	Incubation
**7**	1625.5	0.20	Chick rearing
**8**	165	0.03	Incubation
**8**	129.5	0.45	Chick rearing
**9**	85	0.00	Incubation
**9**	159	0.01	Chick rearing
**0**	397	0.02	Incubation
**0**	616.5	0.64	Chick rearing

^a^ Differences are shown between pair members from eight breeding pairs. Pairs without complete data (both individuals in both periods) were excluded from analyses.

## Discussion

Our study demonstrates that time-lapse photography is a potentially powerful and flexible tool in seabird ecology. It can be used successfully to assess foraging trip parameters and record at-nest behaviours, answering different research questions at the same time. Considering the high manual workload employed and the relatively high failure rates of recording nests in this study, we also make several recommendations for the continued development of this approach.

### Camera failures

Despite the advantages of time-lapse photography, its use must be carefully considered, as shown by the low success rate in terms of nests recorded in our study, obtained despite our best efforts (Table 2). Cameras used for collecting information on foraging and nesting behaviour must be deployed close to nesting sites, with an adequate angle, suitable light conditions and high temporal resolution. The set-up must be done in a way that reveals the presence and the identity of birds at nest in any given time and therefore priority must be given to the correct angle, even if that means a smaller number of nests in the frame. Cameras placed under a cliff facing upwards should be avoided, and we suggest, when possible, to place cameras above a nesting site facing downward at a distance of between 2 and 6 meters. This means that it is always possible to identify the bird at nest. We also noted that it would be possible to capture chick-feeding events, which could be useful in dietary and behavioural studies. In contrast, cameras used for monitoring productivity must be far from the nesting site to maximize the number of nests present in each frame [17] and a lower temporal resolution (e.g. 1 frame per day) can be used. Thus, collecting both data streams simultaneously can prove difficult and the characteristics of the cameras (such as equivalent focal distance) must be considered as well as the temporal resolution used. The camera orientation must be checked in order to avoid having the sun in front of the lenses at any time of the day. The possibility of camera and nest failures must be considered and accounted for with sufficient replication, and therefore regular maintenance and checks of the status of study nests is recommended. We also recommend marking the birds with a distinctive long-lasting dye such as picric acid, since birds marked in green and pink marker pen (excluded from the study) were more difficult to detect in frames with extreme light conditions. Finally, if collecting data on multiple years, we recommend reviewing the first year camera dataset after the first breeding season, in order to ensure that the set-up is correct and there are no gaps in the data due to camera malfunctioning, something which was not possible in the present study.

### Camera data validation

The two measures of foraging trip duration are well correlated, but while biologging devices provide an invaluable method for gaining information on the movement behaviour of seabirds [18], our results demonstrate the potential for misinterpretation of patterns in behavioural data. Observed differences in trip duration between camera and accelerometry data are mainly explained by time spent away from the nest that was not associated with foraging activity: birds were returning to land but not to their nest, leading to an erroneous assignment of nest attendance in accelerometer data. This led to some trips having long durations assigned by camera and short durations assigned by accelerometer, clearly visible as outliers in the lower left corner of Fig 1. This issue highlights both the possibility of behavioural misinterpretation in accelerometry data (such as misidentification of changeovers or periods where the nest is left unattended), and misinterpretation of foraging trip duration recorded with cameras, in the absence of cross-validation. Therefore, foraging trip duration recorded with cameras should not be regarded as an absolute measure of foraging trip duration, and it is perhaps most suitable when used for temporal and/or spatial variability comparisons within or between colonies. Moreover, when testing the presence of a logger effect, foraging trip duration from tagged and untagged birds should be calculated using the same method where possible [41] in order to avoid attributing differences (or absence of differences) resulting from discrepancies between methods to device effects.

### Behaviour at nest

#### Nest attendance

Since kittiwake nests are nearly always attended by one parent, the pattern of nest departures reflects the changeover pattern between partners. Most changeovers in our study were classified by a change in individual birds between frames, rather than by observing both birds at the nest. Due to the low percentage of frames with two individuals present on a nest, we conclude that changeovers nearly always took <4 minutes to complete. As this timeframe corresponds to the temporal resolution of camera data in our study this value only provides an upper estimate. Real changeover times are likely to have been shorter than this. The changeover patterns we recorded indicate that kittiwakes not only partition their foraging effort, but show similar levels of nest attendance. As kittiwakes are known to be largely inactive during night-time [42, 43], the low level of arrivals in the dark suggested that at some stage in the evening, the off-duty birds moved to a roost site rather than returning directly to the nest [44].

#### Predation

Our data suggest that nine of the ten study nests were predated. Predation events recorded in this study in 2013 have previously been reported [45], and predation in subsequent years confirm that at least one peregrine uses this kittiwake colony as a food source during the breeding season. The predicable location of the colony and timing of the kittiwake breeding period perhaps makes this a stable and reliable food source (see S2 Fig for additional evidence of predation events during daylight hours in 2016). We also report for the first time that herring gulls predate on kittiwake chicks at this site (S3 Fig). In the absence of terrestrial predators, avian predation can be an important but underestimated source of breeding failure in colonies of cliff-nesting seabirds [45], and our study supports suggestions that time-lapse photography could aid in identifying and quantifying this pressure [45]. Moreover, analysis of trip durations suggests that, when scared by humans, kittiwakes perform ‘panic flights’ [27] that can be repeated multiple times. In comparison, when scared by an avian predator, they tend to stay away from the nest for longer periods. Unfortunately, sample size of flight following predation attempts was too small to assess if differences in effect were statistically significant. However, the relatively large dataset of trip durations after human presence suggested a mean absence of 20 minutes. This is a useful observation when analysing biologging data, providing a reliable minimum baseline threshold for defining a foraging trip.

### Behaviour away from nest

Previous work on black-legged kittiwakes has shown a negative relationship between foraging trip duration and reproductive performance [10], with declines in breeding success in birds that exhibit longer trip durations, range further from the nest, engage in longer transit times and spent longer rafting on the water [33]. This relationship has been associated with increased rates of egg predation and reduced prey delivery rates as foraging trip parameters increase [29]. Considering the high predation pressure on Puffin Island, from peregrines and gulls, the observed mean trip duration of 4.5 hours in our study has the potential to influence reproductive performance, given suggestions that foraging-related absences from the nest of >5.5 hours during chick rearing may negatively affect breeding success [10] and the fact that in years of high prey abundance in Atlantic and Barents Sea colonies, foraging trips of kittiwakes have been reported to be 2 to 3 hrs in length [42]. In support of this suggestion, productivity on Puffin Island was lower in 2013 and 2014 (0.00 and 0.07 chick/nest, respectively) than during any of the other years from 2010 to 2017 [46]. Further time-lapse photography studies should compare estimates of foraging trip duration between years of high productivity, in order to establish a possible reference threshold for assessing the link between foraging trip duration and reproductive performance.

The observed difference in foraging trip duration between incubation and chick rearing was expected, owing to increasing ties to the nest and constraints on foraging during chick provisioning. However, the use of bio-logging devices is not necessarily sufficient to highlight even such apparently intuitive differences, often due to the short-term deployment that leads to a small number of foraging trip recorded. For example [36] were not able to detect a significant difference in foraging trip duration between incubation and chick rearing using data from same field site and years of study. This demonstrates how the large number of foraging trips provided by time-lapse photography can be used to identify behavioural and ecological patterns that biologging data miss or underestimate. Moreover, time-lapse photography is indispensable for assessing device-biased data. Data collected by the use of bio-logging devices are potentially biased by negative physiological and behavioural effects of these tools on their bearers: prolonged foraging trips could be a result of increased stress or energy requirements associated with device deployment, forcing birds to spend more time at sea to maintain energy reserves [47, 48]. These effects are often poorly considered or not measured at all, in spite of the extensive use of this technology in animal studies [19, 47]. Effects of devices should be measured on all occasions for ethical reasons, but also in order to avoid results being affected by the devices used in a study [41]. Our study suggested that accelerometers did not have a substantial effect on the parameter of kittiwake foraging we recorded (duration). The most substantial reported effects associated with device presence include increase in energy expenditure and a decrease in nest attendance [47]. Therefore, during years of reduced food availability these could lead to a more substantial logger effect. It is therefore particularly valuable that during two years characterized by low productivity at Puffin Island [46] no effect of loggers on nest attendance was detected.

Foraging trip duration on Puffin Island was constant over time both for incubation and for chick rearing periods. Thus there was no evidence of localised prey depletion around the study population, as suggested in other studies of seabirds [49–52], probably due to the current small size of the Puffin Island colony, where the degree of intra and inter specific competition is low. Therefore local prey distribution and abundance is likely to be influenced by oceanographic conditions rather than depletion.

Since foraging trip duration and number of trips per animal did not vary between each pair, it can be concluded that in our study male and female kittiwakes share the same amount of effort in provisioning, both during incubation and chick rearing. This is concordant with the overall findings of [44] and [53] even though in their study colony mean duration for daytime trips by the male was 6% longer than those for the female.

#### Recommendations

Our findings were heavily dependent on marked individuals, which in our case required researcher effort and potential handling stress to animals. Using existing markers, such as plumage features or rings, would be advantageous but technologically challenging. Processing the extensive datasets generated by time-lapse photography requires a high time investment (~90 hours of observer work in this study), and remains one of the major drawbacks to wide-scale use of this technique in ecological studies. Focused citizen science projects could help lightening researchers’ workload [54, 55], however, difficulties in checking images and associated observer biases could lead to questions about data reliability. Furthermore, data processing and validation would remain a time-consuming process. Effort should be given to the development of semi-automated systems for seabird detection in time-lapse photography (such as machine-learning algorithms), as has been done for medium-to large-bodied animals [56]. Such advances will require interdisciplinary collaborations between ecologists and computer scientists, but will enable routine use of these valuable, low cost tools for both conservation and management purposes.

## Supporting information

S1 Fig Frequency of nest departure times of individual kittiwakes from Puffin Island, North Wales. Nest departure frequency per hour during incubation (a) and chick-rearing (b) periods. Unique bird identification codes are given on the y-axis, where nest = first digit and individual = second digit.
(TIF)

S2 Fig Example camera image of diurnal nest predation from peregrine falcon on kittiwake on Puffin Island, North Wales, in 2016. (TIF)

S3 Fig Herring gull predating a kittiwake chick in an unattended nest on Puffin Island, North Wales. Upper panel: kittiwake nest with a chick left unattended from parents. Lower panel: herring gull perched on the nest and predating the chick.
(TIF)

S1 Dataset Duration of all foraging trips by kittiwakes from Puffin Island, North Wales, recorded using time-lapse photography and accelerometers. (XLSX)
