# Supplementary figures and images for: Utility of time-lapse photography in studies of seabird ecology
Source: PLoS One. 2018 Dec 12;13(12):e0208995. doi: 10.1371/journal.pone.0208995 (PMC6291154; doi:10.1371/journal.pone.0208995)

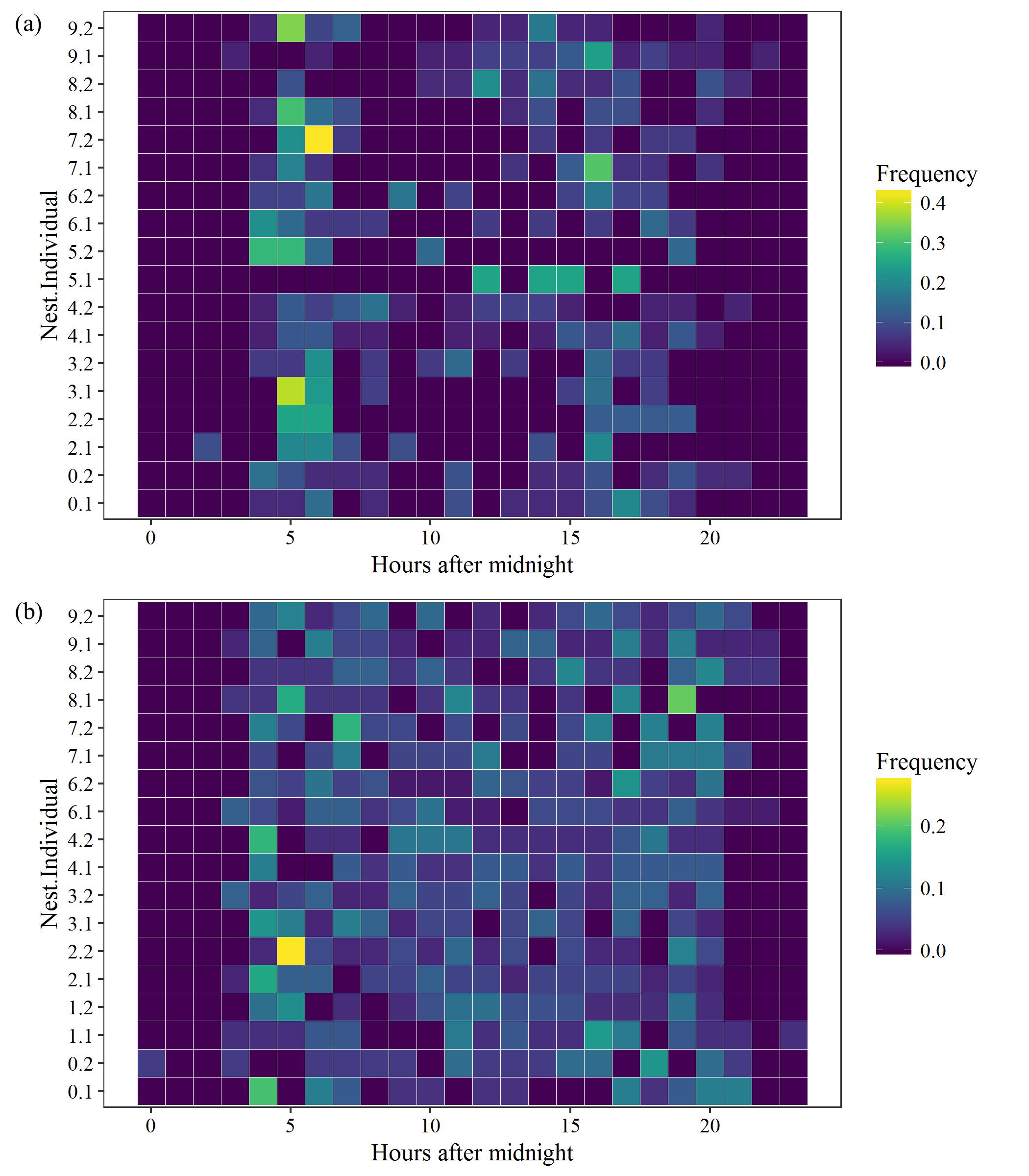

Supplement: S1 Fig — Nest departure frequency per hour during incubation (a) and chick-rearing (b) periods. Unique bird identification codes are given on the y-axis, where nest = first digit and individual = second digit. (TIF) [file pone.0208995.s001.tif]

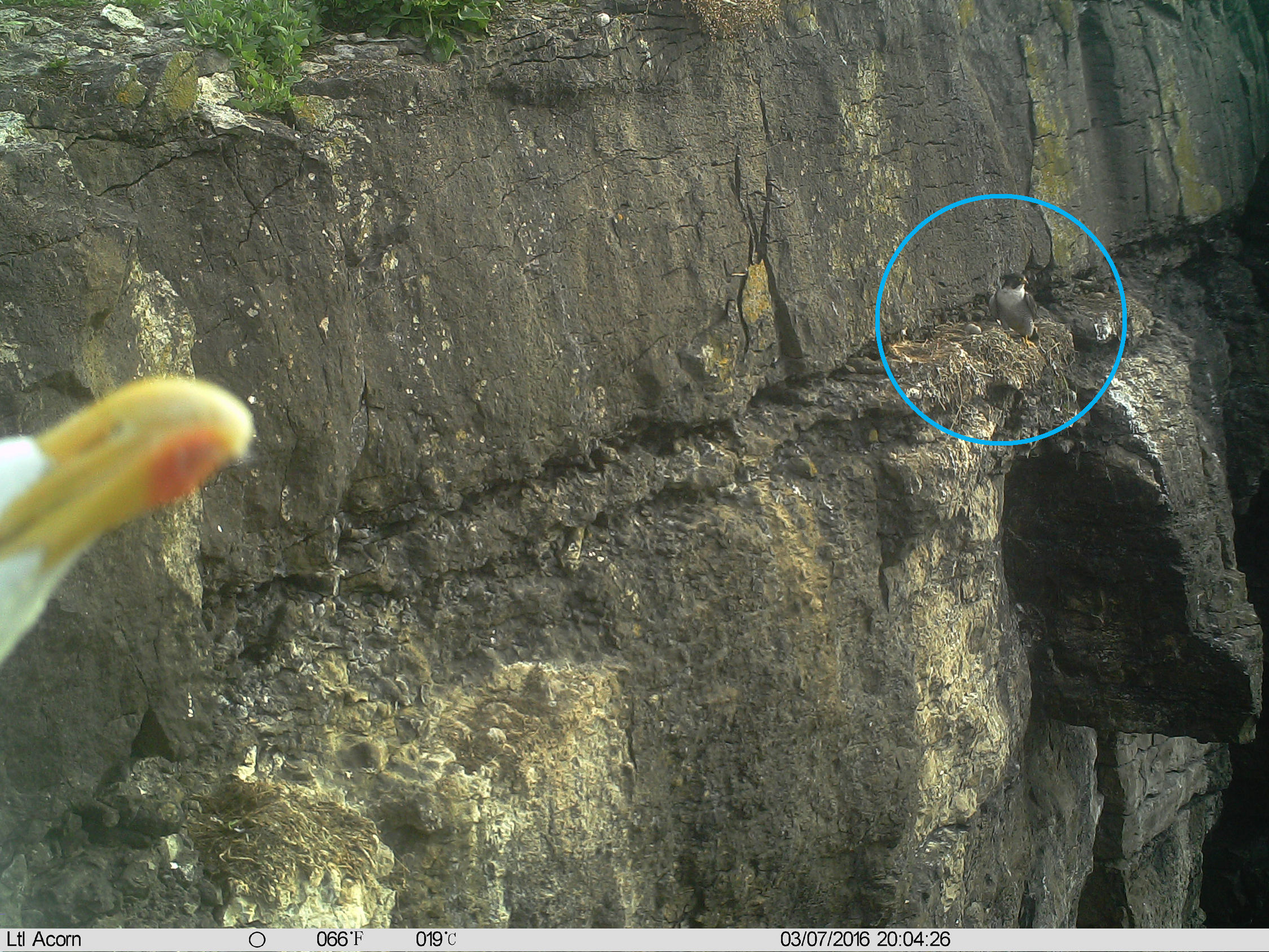

Supplement: S2 Fig — (TIF) [file pone.0208995.s002.tif]

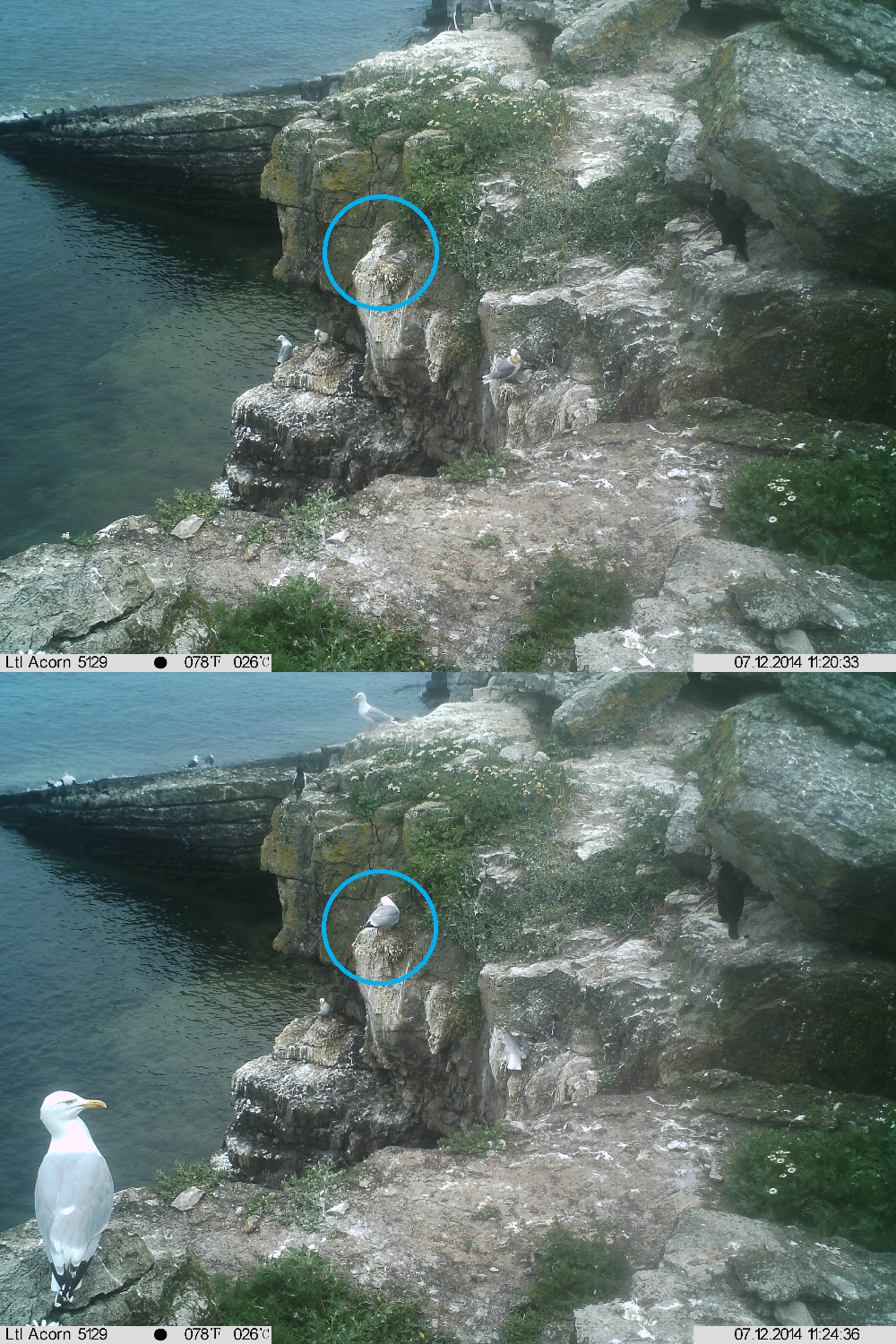

Supplement: S3 Fig — Upper panel: kittiwake nest with a chick left unattended from parents. Lower panel: herring gull perched on the nest and predating the chick. (TIF) [file pone.0208995.s003.tif]
